# Quercetin and aconitine synergistically induces the human cervical carcinoma HeLa cell apoptosis via endoplasmic reticulum (ER) stress pathway

**DOI:** 10.1371/journal.pone.0191062

**Published:** 2018-01-11

**Authors:** Xiu-Mei Li, Jing Liu, Fang-Fang Pan, Dong-Dong Shi, Zhi-Guo Wen, Pei-Long Yang

**Affiliations:** 1 Key Laboratory for Feed Biotechnology of the Ministry of Agriculture, Feed Research Institute, Chinese Academy of Agricultural Sciences, Beijing, China; 2 National Engineering Research Center of Biological Feed, Feed Research Institute, Chinese Academy of Agricultural Sciences, Beijing, China; 3 Key Lab of Industrial Fermentation Microbiology of the Ministry of Education, Tianjin Key Lab of Industrial Microbiology, College of Biotechnology, Tianjin University of Science and Technology, Tianjin, China; National Cheng Kung University, TAIWAN

## Abstract

Up till now, studies have not been conducted on how the combination of Quercetin (Q), Aconitine (A) and apoptosis induction affects human cervical carcinoma HeLa cells. The result of our findings shows that the combination of Q and A (QA) is capable of synergistically inhibiting the proliferation of HeLa cells in a number of concentrations. QA synergistically inhibits the proliferation of MDR1 gene in the HeLa cells. It is concluded based on our result that QA induces apoptosis and ER stress just as QA-induced ER stress pathway may mediate apoptosis by upregulating mRNA expression levels of eIF2α, ATF4, IRE1, XBP1, ATF6, PERK and CHOP in the HeLa cells. The up-regulating of mRNA expression level of GRP78 and activation of UPR are a molecular basis of QA-induced ER stress.

## Introduction

Cervical cancer is one of the leading causes of death among women worldwide [1]. Approximately 85% of cervical cancer cases occur in low- and middle-income countries [2]. While there are established methods of treatment for affected people, such methods possess significant side effects. Natural treatment products, on the other hand, or phytochemicals, are preferred for their safety and lower drug resistance [3]. Developing effective anti-cancer drugs from plants require great effort. Over 60% of anticancer drugs have their main ingredients from natural sources [4]. Herbal medicines fight cancer by inhibiting cell proliferation, inducing apoptosis and modulating cell signaling pathway and oxidative stress-mediated mechanism [5, 6].

Quercetin (Q), a flavonoid found in fruits, vegetables, tea and other plant-derived foods, has unique biological properties capable of improving mental/physical performance and reducing infection risk [7,8]. Q has been found to have anti-proliferative and pro-apoptotic effects on HeLa cells [9]. It is active throughout the stages of carcinogenesis, from initiation to invasion and metastasis, and is able also to undermine the basis of the development and maintenance of tumors. PI3K/Akt pathway in HeLa cells have been found to mainly mediate the anti-proliferative and apoptotic processes caused by Q.

Aconitine (A), which is also known as monkshood or devil’s helmet, is a kind of Aconitum plant-produced toxin [10]. A is often used as the antipyretic and analgesic agent in many Asian countries in spite of its cardiotoxic and neurotoxic risks. However, it is difficult to measure its appropriate dosage due to its narrow therapeutic index [11, 12].

The failure of chemotherapy-based treatment has been attributed largely to Multidrug resistance (MDR), the development of which has been linked to chemotherapeutic failure in cancer treatment. An MDR phenotype may occur from mechanisms such as alteration of drug targets, increased DNA repair, reduced cell apoptosis and overexpression of drug efflux transporters [13]. Some studies showed that overexpression of drug efflux proteins in the ATP-binding cassette superfamily, particularly P-glycoprotein (P-gp; encoded by *MDR1* or *ABCB1* gene), inhibited the intracellular accumulation of cytotoxic drugs, causing a reduction of drug-mediated cytotoxicity [14]. The cells ultimately develop resistance toward these cytotoxic drugs.

Apoptosis is a preferred method for cancer chemotherapy [15]. As suggested by recent studies, ER stress also contributes to apoptosis just as ER stress-induced apoptosis pathways have become a popular field of research [16]. The ER accurately ensures proteins are folded and assembled before they are sent to other organelles [17, 18]. ER stress occurs when abnormalities such as protein misfolding and unfolded protein accumulate in the ER lumen, resulting in the dissociation of GRP78 from the ER stress transducers and triggering the activation of the UPR branches [19–22]. ER stress-induced apoptosis can occur when UPR fails to compensate for the abnormalities [23].

In summary, the objective of this study was to evaluate the effect of Q and A in combination with apoptosis induction via endoplasmic reticulum (ER) stress pathway in human cervical carcinoma HeLa cells.

The mRNA expression of GRP78, which was the maker of ER stress was determined. The mRNA expression of UPR pathway (eIF2α, ATF4, IRE1, XBP1, ATF6, PERK and CHOP) was also measured.

## Materials and methods

### Chemicals and reagents

Quercetin and Aconitine were purchased from the National Institutes for Food and Drug Control (Beijing, China).

### Cell culture

Human cervical carcinoma HeLa cells were purchased from the American Type Culture Collection (ATCC) and cultured in DMEM/F12 containing 10% FBS, 100 μg/mL streptomycin and 100 units/mL penicillin at 37°C in a controlled condition at 5% CO_2_ and 90% relative humidity.

### Cell viability

Cell viability effect of drug on HeLa cells was determined, with some modifications, by MTT assay as previously described [24]. HeLa cells were seeded in 96-well plates (10^5^cells/well) and cultured overnight. Next, the cells were treated for 48hrs using different drug concentrations. After treatment, the cells were washed twice with phosphate buffered saline (PBS) and incubated with 20 μL MTT (5mg/mL) and 80 μL DMEM for 4 hrs. MTT solution was then removed and 100 μL DMSO was added to each well. The absorbance was measured at 490 nm by Microplatereader. All the results were expressed as the inhibition ratio of cell proliferation calculated as [(A-B)/A] ×100%, where A and B are the average numbers of viable bacterial cells of the control and samples, respectively.

### Computation of the combination index for quantitative determination of drug interactions

In drug combination studies, many researchers are interested in quantifying drug interactions and classifying the interactions into categories of synergy, additivity, or antagonism [25]. The most widely used method for evaluating drug interactions in combination cancer chemotherapy is Combination Index (CI) analyses [25, 26]. The Loewe additivity model has been a reference model majorly used when the combined effect of two drugs is additive [25, 26]. The model can be written as in Eq (1):

$$\frac{(D)1}{(\mathrm{Dx})1}+\frac{(D)2}{(\mathrm{Dx})2}=1$$ (1)

where (D)_1_ and (D)_2_ are the respective combination doses of drug 1 and drug 2 that yield an effect of 50% growth inhibition, with (Dx)_1_ and (Dx)_2_ being the corresponding single doses of drug 1 and drug 2 that result in the same effect, which is by definition the GI50 of drug 1 and drug 2. When Eq 1 holds, it can be concluded that the combined effect of the two drugs is additive. Based on Eq 1, the combination index, defined in Eq (2), can be used to classify drug interactions as synergistic, additive, or antagonistic [25, 26].

$$\mathrm{CI}=\frac{(D)1}{(\mathrm{Dx})1}+\frac{(D)2}{(\mathrm{Dx})2}$$ (2)

CI<1 synergy; CI = 1 additivity; CI>1 antagonism

A CI of less than, equal to or more than 1 indicates synergy, additivity or antagonism, respectively. We simulated isobolograms for a pair of drugs with eight equally effective dose combinations for a particular effect level of GI50. GI50 normalized doses of drug 1 and drug 2 that give this effect in combination are plotted as axial points in the isobologram graphs. According to Eq 2, the isobologram curves are expected to be parallel to the diagonal for additive drug pairs, concave for synergistic drug pairs, and convex for antagonistic drug pairs. We focused on the qualitative shape of the isobolograms to correctly identify the drug pair category, and use the smallest CI of the eight drug dose combinations as the CI for this drug pair [25, 26].

### Measurement of reactive oxygen species

According to manufacturesʹ instructions, the level of reactive oxygen species (ROS) was measured by dichloro-dihydro-fluorescein diacetate. Briefly, cells were incubated with dichloro-dihydro-fluorescein diacetate (final concentration of 10 μM) at 37°C in the dark for 30 minutes, then washed with cold phosphate-buffered saline (PBS), and then analyzed immediately, using a FACS.

### Cell apoptosis assay

The FITC Annexin-V/Dead Cell Apoptosis Kit with FITC annexin V and propidium iodide (PI) (Invitrogen, Molecular Probes, USA) for flow cytometry provides a rapid and convenient assay for apoptosis. In this protocol, 1×10^5^ cells were seeded and washed twice with PBS, and then mixed with 100 μL binding buffer to form a cell suspension. FITC annexin-V 5μL was mixed with 1 μL of 100 μg/mL PI working solution. The cells were incubated in the dark for 15 minutes at room temperature. 400 μL binding buffer was then added, mixed gently and analyzed immediately using an FACS.

### TUNEL assay

We used in situ cell death detection kit (molecular probes, Invitrogen, USA). The method distinguishes apoptotic cells from those undergoing necrosis because damaged DNA in the former leads to a different distribution of staining and nuclear morphology. Firstly, the testis sections were fixed for one hour using 4% paraformaldehyde (PFA) and later washed three times with PBS. The sections were then submerged in PBS with 0.1% Triton-X-100 in an ice bath for 2 min. After further washes with PBS, the reaction was performed in the terminal deoxynucleotidyl transferase (TdT) buffer with fluorescein labeled dUTP. The samples were then incubated with a reagent for one hour at 37°C with plastic membrane mounted to avoid reagent evaporation. Washed with PBS, the sections were incubated with a mixture of DAPI and antifade reagent before coverglass mounting. A positive TUNEL preparation kit (Beyotime, China) was used to prepare the positive control. The sections were treated with DNase I for 30 min at 25° and PBS wash before TUNEL labeling reaction. The TUNEL results were then analyzed with a Leica scanning confocal microscope (TCS SP5) (Leica, Germany).

### Mitochondrial membrane potential (*Δψm*) assay

Flow cytometry (Bio-Rad, USA) and mitochondria-selective dye JC-1 detection kit (Molecular Probes, USA) were used to measure the mitochondrial membrane potential (***Δ****ψ*_*m*_). Mitochondrial uptake of JC-1 is dependent on its lipophilic action driven by the polarity of ***Δ****ψ*_*m*_. In normal mitochondria, JC-1 exists as a polymer emitting red fluorescence that can be detected by flow cytometry FL-2 channel. If ***Δ****ψ*_*m*_ is depolarized, the JC-1 enters the mitochondria and presents as monomer green fluorescence that can be detected by FL-1 channel. The JC-1 was used to detect the mitochondrial membrane potential, an indicator of mitochondrial dysfunction and early apoptosis.

A mitochondrial membrane potential assay kit was used to detect the potential loss of mitochondrial membrane. Briefly, cells were seeded in 6-well plates at a density of 1×10^5^ cells/mL and cultured overnight, then treated with QA for 24hrs. The cells were twice washed with PBS then stained with JC-1 for 20 min at 37°C with 5% CO_2_-95% air. Then the cells were twice washed with JC-1 staining buffer and analyzed immediately by FCM.

### Real time polymerase chain reaction (RT-PCR)

HeLa cells were plated into six well plates at a density of 1×10^5^ cells per well. Cells were treated with QA (44.08 μg/mL) for 24hrs after 12hrs of cultivation. Total RNA was then isolated by using Trizol reagent (Invitrogen, Carlsbad, USA). First-strand cDNA was synthesized with random hexamer primers. PCR primers specific for MDR1, IRE1α, PERK, XBP1, ATF4, eIF2α, GRP78, ATF6, CHOP or GAPDH, the internal control, were listed in **S1 Table**. Briefly, the amplification of primer was carried out with 40 cycles at a melting temperature of 94° for 15secs, an annealing temperature of 60° for 1 min, and an extension temperature of 72° for 50 s. The fold or percentage of change in the relative expression of the mRNA of the target gene was measured by the 2^-△△Ct^ method.

### Statistical analysis

All the data were expressed as mean±standard deviation. The statistical analysis was performed using one-way analysis of variance test for multiple comparisons. The differences between comparisons were considered to be statistically significant at *P*<0.05. SPSS software version 17.0 (SPSS Inc., Chicago, IL, USA) was used for data analysis.

## Results and discussion

### How the growth inhibition of HeLa cells is induced by the combined treatment of Q and A

**Fig 1:**
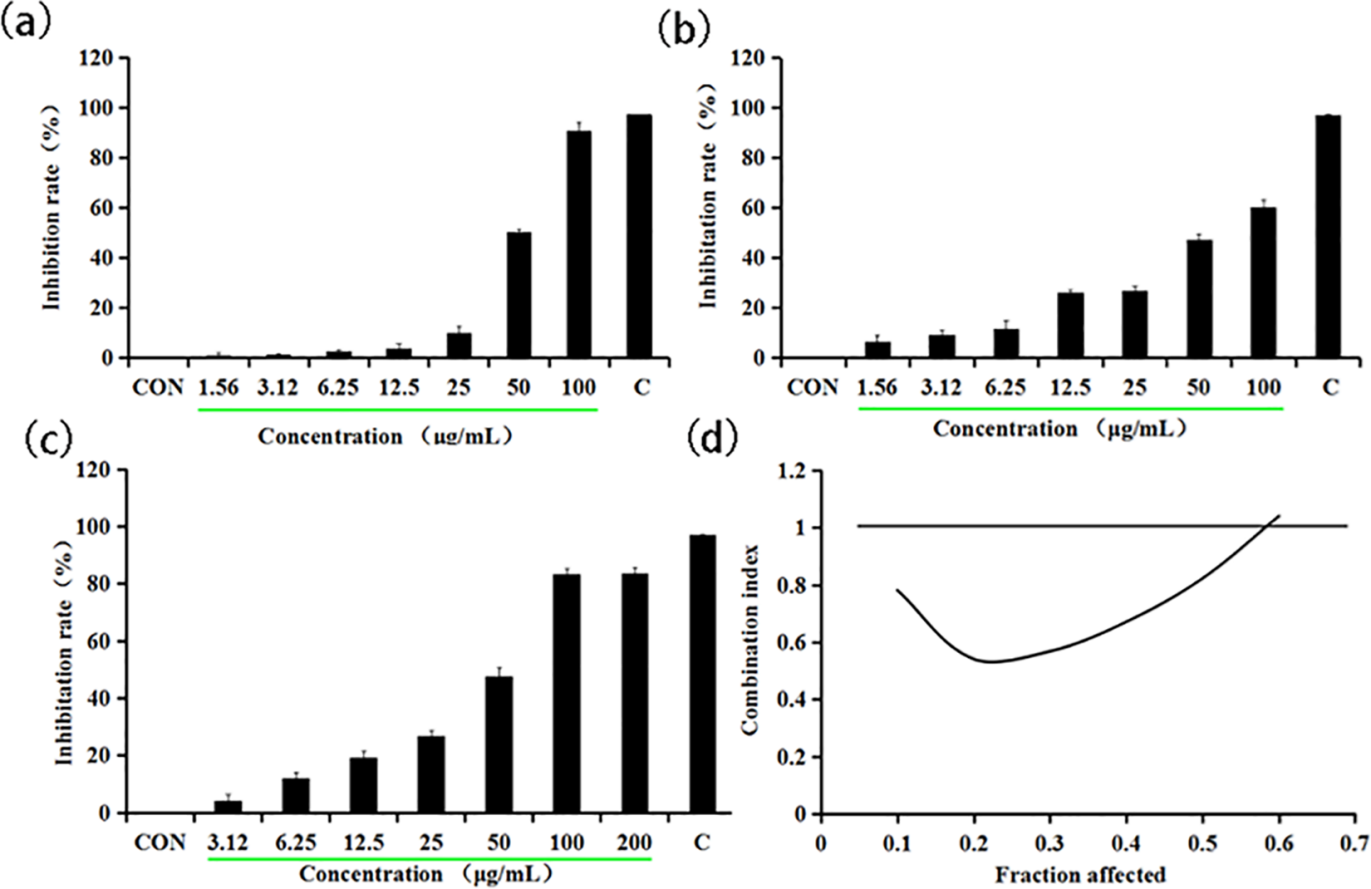
Quercetin combined with aconitine inhibit Hela cell proliferation. (a) Quercetin inhibits Hela cell proliferation. (b) Aconitine inhibits Hela cells proliferation. (c) Quercetin combined with aconitine inhibit Hela cells proliferation. (d) Median effect analysis of curcumin in combination with Q and A.

First, we assessed the effect of Q or A as a single agent in the growth of the HeLa cells. Either Q or A is able to individually cause a markedly dose-dependent reduction in cell viability, with 50% growth inhibition (IC_50_) of 55.99 μg/mL (**Fig 1A)** and 71.89 μg/mL (**Fig 1B**), respectively. Next, we attempt to estimate the combined effect of Q and A on HeLa cell viability by adopting a combination treatment, using varying concentrations of Q (0–100 μg/mL), together with varying concentrations of A (0–100 μg/mL) at 1:1 concentration ratio. In the end, the combined treatment of Q and A substantially inhibited HeLa cell growth as against single drug alone. **Fig 1C** showed 0.78-fold and 0.61-fold decrease of IC_50_ compared to Q or A treatment alone, indicating that the combination treatment with Q and A is more effective in inhibiting the growth of HeLa cells than either Q or A alone.

To evaluate their synergistic effect on HeLa cells, we assessed synergy using CalcuSyn software to evaluate the combination index originally described by Chou and Talalay, where synergism, additivity and antagonism are defined as CI<1, CI = 1 and CI>1, respectively [25]. The CI value was nearly 0.6 when HeLa cells were exposed to the combination of 61.75 μg/mL QA, indicating a slight antagonism (**Fig 1D**). However, HeLa cells, upon exposure to the combination of variable concentrations of QA (0–61.75 μg/mL), demonstrated clear evidence of synergy since the CI value ranged from 0.1 to 0.6 (**Fig 1D**).

Q, found in the plants, such as vegetables and fruits, is becoming a major field of research because of its potential health benefits and minimal side effects [9]. It has been reported to have the antiproliferative and proapoptotic effects on HeLa cells. It is also an anti-tumor agent, which works on virtually all the stages of carcinogenesis and can undermine the basis of the development and maintenance of tumors [9]. A, also known as monkshood or devil’s helmet, is an Aconitum plant-produced toxin. It is commonly used as a traditional medicine in China because of its analgesic and anti-inflammatory activities, in spite of its narrow therapeutic index.

### QA synergistically inhibits the proliferation by MDR1 gene in the HeLa cells

**Fig 2:**
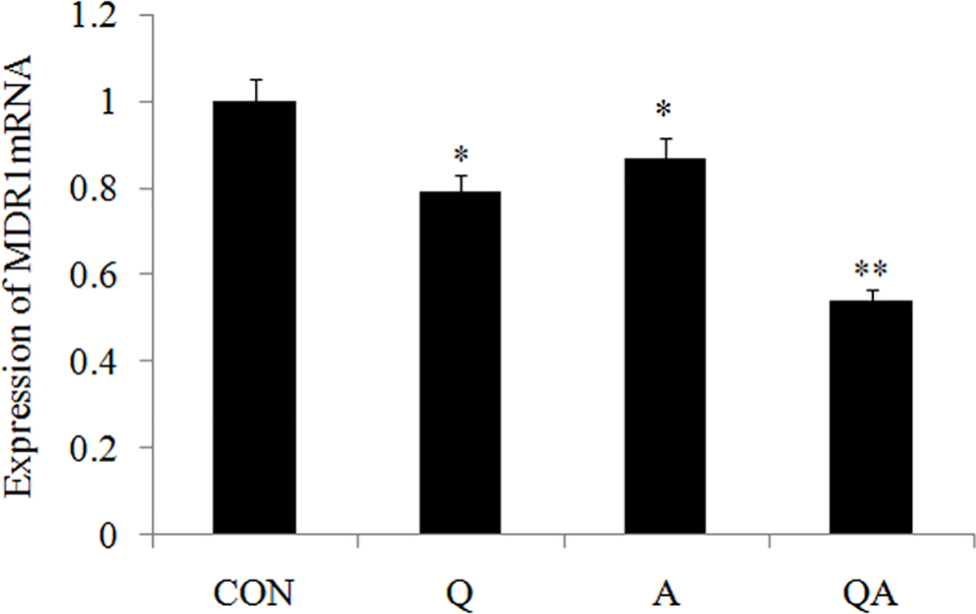
(a-c) RT-PCR was performed to examine the effect of Q, A and QA on the mRNA expression of MDR1. GAPDH served as an internal control. Data are presented with mean±standard deviation (n = 5) *p<0.05, compared with the control group; *p<0.01, compared with the control group.

The failure of chemotherapy-based treatment occurs majorly because of MDR. Of the many mechanisms of MDR, the high expression of the human MDR1 gene and the P-glycoprotein (P-gp) transporter encoded by MDR1 attract the highest level of scrutiny among researchers [27]. Tumor cells that overexpress MDR1/P-gp usually show resistance to various chemotherapeutics. From **Fig 2**, we see that all of the results showed that the expression of MDR1 mRNA level decreased in the Q (0.79±0.03), A (0.85±0.04) and QA (0.54±0.02) groups, whereas the QA (0.54±0.02) group showed a better effect. Furthermore, previous study have shown that Q inhibits the expression of P-gp. It is able to reduce the degradation rate of the drug in the body and the cells, so as to improve the intake rate of tumor tissue on drug [28].

### QA induces HeLa cell apoptosis

#### Reactive oxygen species

**Fig 3:**
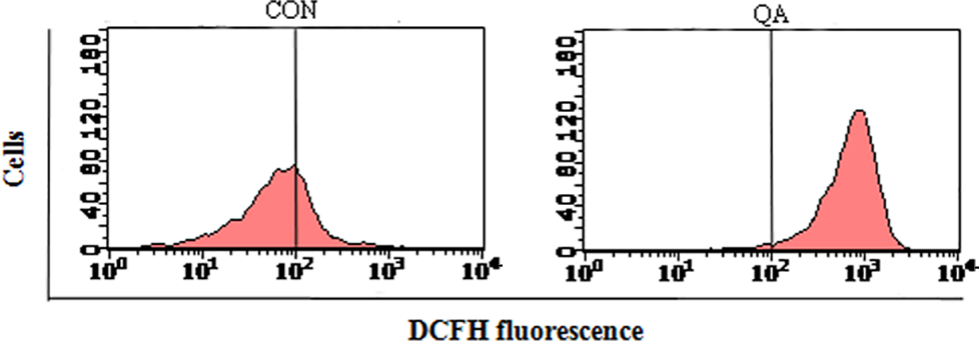
Effect of QA on the intracellular ROS generation in HeLa cells. Cells were stained using DCHF2-DA and then analyzed using a flow cytometer.

ROS generation is one of the events that take place at the onset of apoptosis [29]. As shown in **Fig 3**, incubating HeLa cells with QA (44.08μg/mL) for 24 hrs at 37°C caused a significant increase in DCF fluorescence. It is suggested that ROS is generated by many anticancer drugs by causing oxidative stress and inducing apoptosis in cancer cells, while many inhibitors of apoptosis have antioxidant activity [30]. Indeed, factors that cause or promote oxidative stress, such as ROS production, lipid peroxidation and the down-regulation of antioxidant genes, are involved in apoptotic processes [31]. By inducing mitochondrial membrane damage, ROS induction up-regulates the activity of certain enzymes that are involved in the cell-death pathway [29]. With these results, we established that QA treatment induced the growth inhibition and ROS generation in HeLa cells, indicating that ROS production was the likely cause of QA-induced apoptosis.

#### Cell apoptosis assay

**Fig 4:**
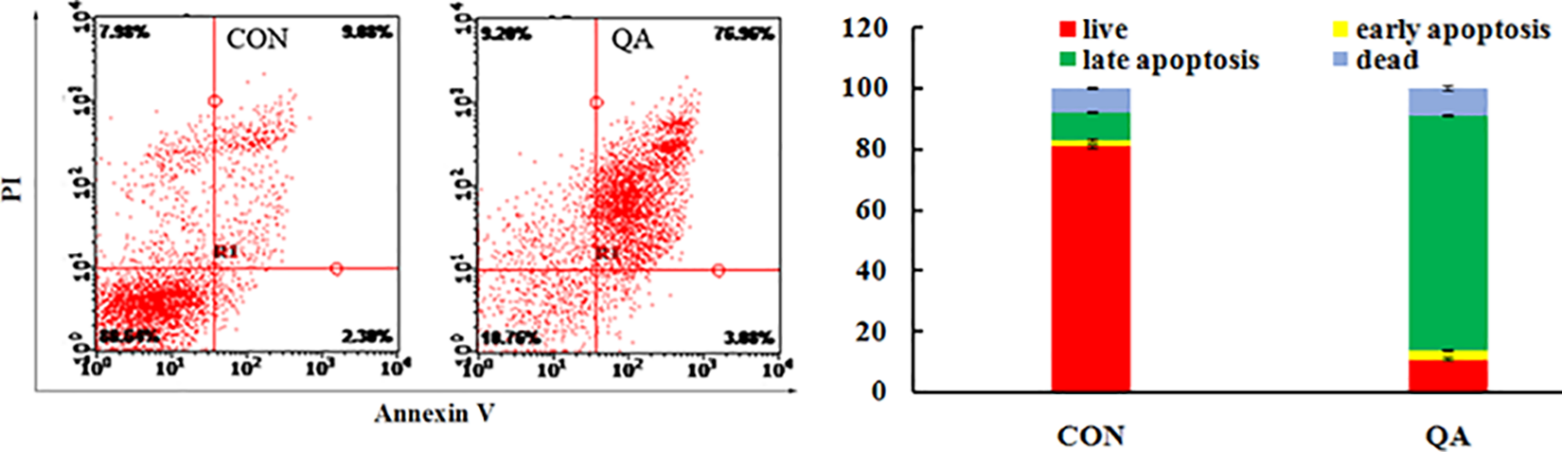
Analysis of cell apoptosis. (a) Flow cytometric measure of PS externalization in HeLa cells. (b) Representation of the frequency of live, early apoptotic, late apoptotic and necrotic cells from (a).

Annexin-V/PI double staining was used to differentiate intact cells from early apoptotic cells, late apoptotic cells, and dead (necrotic) cells as well as to investigate apoptosis in greater detail [32]. **Fig 4** quantifies the increase in apoptotic cell labeling with Annexin V^+^ /PI^-^, which increases from 11.38% in the control group to 80.04% in the QA-treated group. Taken together, these observations suggested that QA significantly stimulated apoptosis in HeLa cells.

#### TUNEL assay

**Fig 5:**
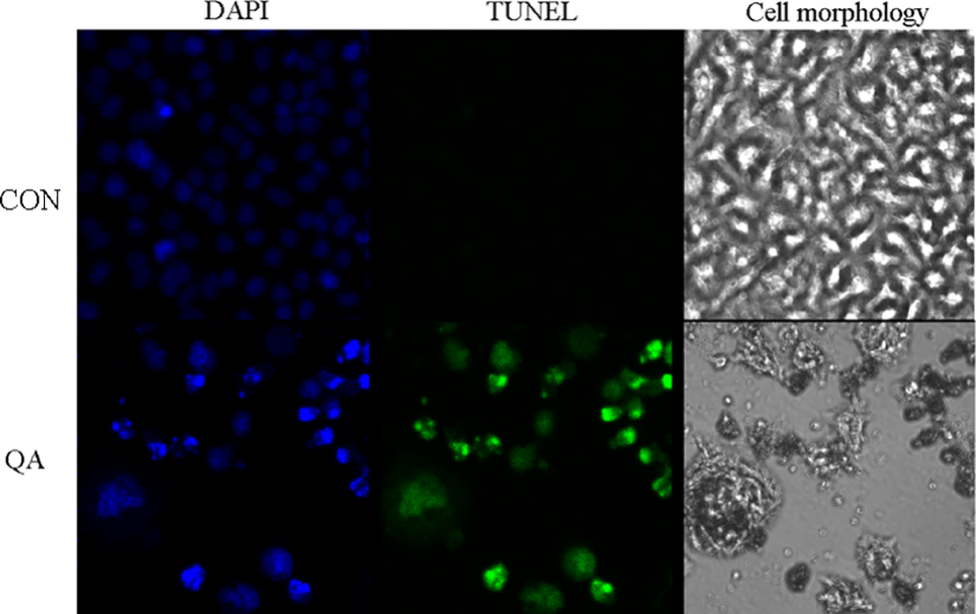
DNA and nuclear damage by QA was measured by fluorescence microscopy using TUNEL and DAPI staining in cells.

The TUNEL assay can detect DNA strand breaks that are mainly induced by reactive oxygen species (ROS) and abortive apoptosis. To confirm that QA is connected to late apoptosis in HeLa cells, DNA damage and nuclear fragmentation in HeLa cells were analyzed by TUNEL and DAPI staining, respectively (**Fig 5**). Through confocal microscopy observation, the cells with QA showed a strong green fluorescence or green fluorescent spots, which indicated DNA fragmentation (**Fig 5**). These findings were further corroborated with those of DAPI staining, as DAPI binds to AT sites on the minor groove of DNA and emits fluorescence. Similarly, it was found that HeLa cells exposed to QA had a DAPI-positive phenotype and showed chromatin condensation, indicating nuclear fragmentation. We established from our results that QA induced changes in the structure and content of the nuclear DNA of HeLa cells (**Fig 5**).

#### Mitochondrial membrane potential

**Fig 6:**
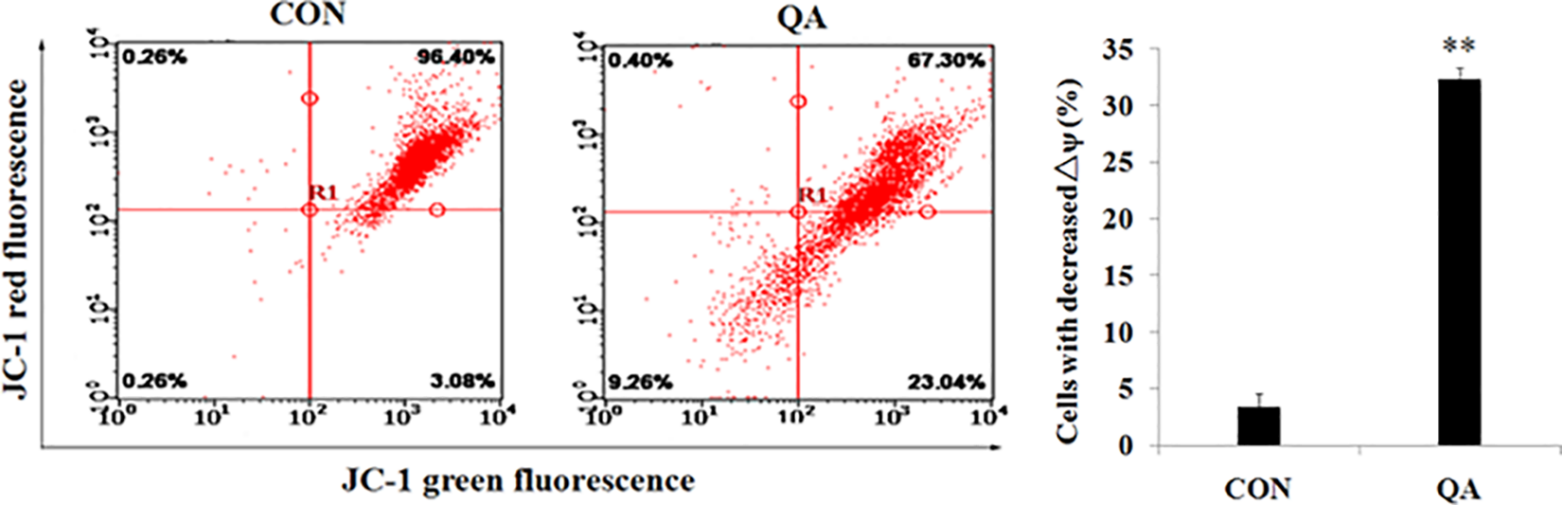
(a,b) Effect of QA on the *Δψm* in HeLa cells. Cells were stained using JC-1 and then analyzed using a flow cytometer.

Mitochondrial membrane is usually damaged by excess ROS generation and this can lead to cell death [33, 34]. Mitochondrial dysfunction, including the loss of mitochondrial membrane potential (*ΔΨm*) and the release of cytochrome c from the mitochondria into the cytosol, is associated with apoptosis [35]. We, therefore, examined whether QA-induced ROS generation could trigger mitochondrial membrane damage in HeLa cells. After being treated with QA (44.08 μg/mL) for 24 hours, HeLa cells were stained with a membrane potential indicator JC-1. The mitochondrial membrane potential was then analyzed by FCM. QA treatment resulted in a significant loss of mitochondrial membrane potential, as shown in **Fig 6**, indicating that QA can induce mitochondrial dysfunction from 3.08±0.62% to 23.04±1.14% in HeLa cells. These data suggested that the augment of intracellular ROS generation induced by QA might be an upstream event of mitochondrial membrane potential.

#### QA induces ER stress by activating UPR pathways in the HeLa cells

**Fig 7:**
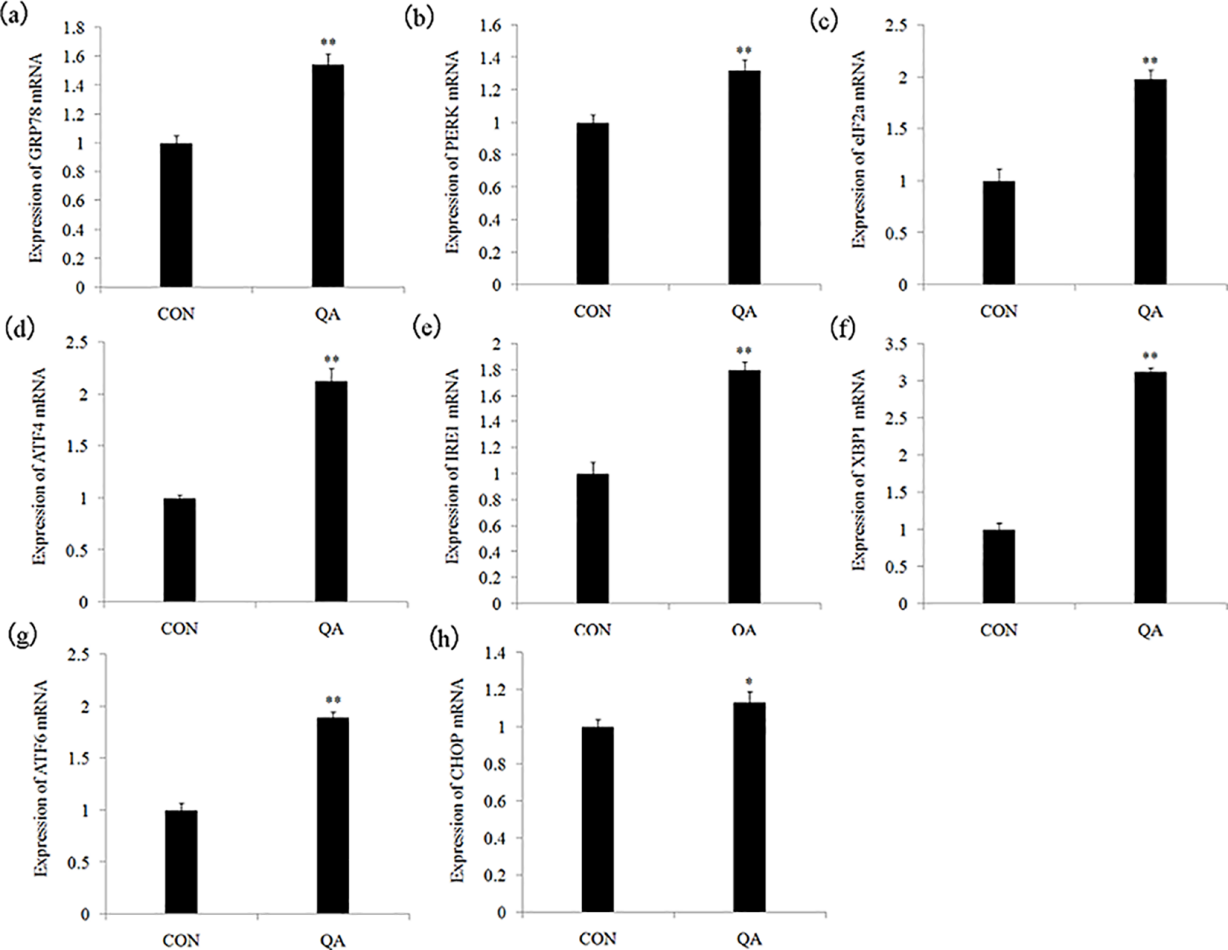
(a-h) RT-PCR was performed to examine the effect of QA on the mRNA expression of GRP78, IRE1α, PERK, XBP1, ATF4, eIF2α, ATF6, CHOP in HeLa cells. GAPDH served as an internal control. Data are presented with mean±standard deviation (n = 5) *p<0.05, compared with the control group; *p<0.01, compared with the control group.

It has been demonstrated by some studies that an ER chaperone protein GRP78 serves as a master UPR regulator and plays essential roles in activating PERK, IRE1, and ATF6, in response to ER stress [36]. GRP78, also known as BiP, is a multi-functional protein predominantly expressed in the lumen of the ER. Typically, GRP78 acts as a major ER chaperone and a master regulator of ER stress signaling through controlling protein folding and assembly, preventing protein aggregation, and regulating signaling of the unfolded protein response [37–43]. First, we examined whether QA could induce ER stress in the HeLa cells. We examined the mRNA expression of GRP78, which was the marker of ER stress. As shown in **Fig 7A**, GRP78 mRNA expression was significantly higher (*p* < 0.05 or *p*< 0.01) in the QA-treated group (1.58±0.09) than it was in the control group (1.00±0.04). We examined all the three UPR pathways-PERK pathway, IRE1 pathway and ATF6 pathway-to further confirm that UPR pathways were involved in QA-induced ER stress. In **Fig 7B**–**7H**, eIF2α (1.95±0.08), ATF4 (2.16±0.10), IRE1 (1.81±0.07), XBP1 (3.23±0.02), ATF6 (1.82±0.01), PERK (1.31±0.09) and CHOP (1.11±0.05) mRNA expression was significantly higher (*p*< 0.05 or *p*< 0.01) in the QA groups than that in the control group (1.00±0.04).

UPR is a defense mechanism against various cellular stress causing accumulation of unfolded proteins in the ER [44]. The chaperone protein GRP78 is a major regulator of all three UPR pathways-PERK pathway, IRE1 pathway and ATF6 pathway-which we also monitored. Under physiological conditions, the luminal domains of PERK, IRE1 and ATF6 proteins are kept inactive when bound to the ER resident chaperone GRP78 [45]. With the accumulation of unfolded proteins, GRP78 releases enable PERK dimerization and activation to phosphorylate eIF2a. The phosphorylated eIF2a then induces the translation of ATF4 mRNA [46]. In this study, the results showed that QA increased the eIF2a and ATF4 mRNA expression, which means that PERK pathway, is one of the mechanisms of QA-induced ER stress. ATF4 promotes many adaptive responses that restore ER function and maintain cell survival [47]. It also promotes apoptosis by regulating the CHOP and Noxa [48]. It is found that QA also activated IRE1 pathway, which was characterized by increasing the IREI and XBP1 mRNA expression. GRP78 is released from IRE1 and permitted to dimerize activating XBP1 kinase and RNase activities to initiate XBP1 mRNA splice. This produces a potent transcriptional activator [49]. The XBP1 mRNA expression levels are increased in QA-treated HeLa cells. Concurrently, the increase of ATF6 mRNA expression showed that QA activated the ATF6 pathway [50].

## Conclusions

Based on our findings, we conclude that Q combined with A synergistically inhibits the proliferation of HeLa cells in a wide range of concentrations. QA synergistically inhibits the proliferation by the MDR1 gene in the HeLa cells. Our results suggest that QA induces apoptosis and ER stress just as QA-induced ER stress pathway may mediate apoptosis by upregulating mRNA expression levels of eIF2α, ATF4, IRE1, XBP1, ATF6, PERK and CHOP in the HeLa cells. The upregulating of the mRNA expression level of GRP78 and the activation of UPR are a molecular basis of QA-induced ER stress.

## Supporting information

S1 Table Primers used for RT-PCR. (DOC)
(DOC)
