# Supplementary material for: Quercetin and aconitine synergistically induces the human cervical carcinoma HeLa cell apoptosis via endoplasmic reticulum (ER) stress pathway
Source: PLoS One. 2018 Jan 11;13(1):e0191062. doi: 10.1371/journal.pone.0191062 (PMC5764366; doi:10.1371/journal.pone.0191062)
Supplement: S1 Table — (DOC) (DOC) [file pone.0191062.s001.doc]

| Gene | Foward Primer(5′→3′) | Rwverse Primer(5′→3′) |
| --- | --- | --- |
| MDR1 | GGAGGACAAATGGAGGAC | GCACCGAGTATGAGGACA |
| GAPDH | AGGGCTGCTTTTAACTCTGGT | CCCCACTTGATTTTGGAGGGA |
| IRE1α | TTGGGCCAGTACTTTTGACC | GGGATGTCCTCCTTCCTCTC |
| PERK | ATGATCATTCCTTCCCTGGAT | AGTCAGAGATTTTCCTCCAACC |
| XBP1 | AAGCCAAGTGAGTACTGGTTCC | GATTGCATGGTAATCTTGTTGG |
| ATF4 | AACAACAGCAAGGAGGATGC | GGGGCAAAGAGATCACAAGT |
| eIF2α | TCCGAGGATCAGAAGGACTG | TGTTCAAAATACACCACCAACA |
| GRP78 | CCACCTCAGTCTCCCAGCTAA | GCCGAGCATGGTGGTAACA |
| ATF6 | ACCCACTAAAGGCCAGACG | CCACGTGATTAGGGAGCTGT |
| CHOP | AAGGCACTGAGCGTATCATGT | TTTATGGCTGCTTTGGTGCT |

**Table 1** Primers used for RT-PCR
